# Moving CtDNA from prognosis to practice in esophageal cancer: challenges and opportunities – Letter to the Editor “circulating tumor DNA as a biomarker for progression and survival in esophageal cancer after neoadjuvant therapy and esophagectomy: a systematic review and meta-analysis”

**DOI:** 10.1097/JS9.0000000000003395

**Published:** 2025-09-08

**Authors:** Man Sun, Dan Zang, Jun Chen

**Affiliations:** a Department of Oncology, The Second Hospital of Dalian Medical University, Dalian, Liaoning, China

*To the editor*

We read with interest the recent meta-analysis by Shen *et al*^[1]^, which synthesized current evidence on the prognostic value of circulating tumor DNA (ctDNA) after neoadjuvant therapy and surgery in esophageal cancer. As ctDNA gains traction across solid tumors, its clinical utility in perioperative decision-making remains underdefined – despite growing interest in biomarker-guided surveillance and adjuvant strategies. Here, we outline key limitations that must be addressed to realize its integration into surgical oncology practice. This study complies with the TITAN 2025 guidelines for transparent and ethical reporting of AI-assisted research^[2]^.

First, while the meta-analysis convincingly demonstrates the association between postoperative ctDNA positivity and poor survival in esophageal cancer, it leaves a pivotal translational question unanswered: how should ctDNA guide clinical decisions? Despite statistical rigor, the authors provide no evidence on whether ctDNA-positive patients received tailored interventions, nor do they propose actionable thresholds or response-adaptive frameworks. In the absence of predefined triggers or escalation algorithms, ctDNA remains a retrospective correlate, not a prospective tool. This gap is especially pressing given the success of ctDNA-informed trials such as DYNAMIC (colon cancer) and TRACERx (lung cancer), which have catalyzed paradigm shifts toward molecularly guided treatment^[3,4]^. Without clarifying how ctDNA status modifies postoperative pathways, the study risks reinforcing the prevailing skepticism around its utility in real-world surgical oncology.

Second, reducing ctDNA to a binary variable – positive versus negative – oversimplifies a biologically continuous marker, risking misclassification and obscuring prognostic gradients. Quantitative parameters such as variant allele frequency, clearance kinetics, and longitudinal dynamics outperform static detection in stratifying recurrence risk and minimal residual disease^[5]^. By collapsing genomic heterogeneity into dichotomous endpoints, the meta-analysis precludes dose–response modeling and diverges from emerging standards in breast, lung, and colorectal cancers, where ctDNA is increasingly used as a dynamic surrogate^[6]^. Future studies must adopt ctDNA as a temporal, quantitative biomarker reflecting real-time tumor evolution and therapeutic pressure.

Third, although some included studies incorporated immune checkpoint inhibitors (ICIs) into neoadjuvant regimens, the meta-analysis failed to stratify ctDNA dynamics within immunotherapy-treated cohorts. This omission is nontrivial: ctDNA kinetics under ICIs differ markedly from those seen with cytotoxic therapies, with phenomena such as pseudoprogression, delayed clearance, or immune-driven fluctuations potentially confounding interpretation. In NSCLC and melanoma, ctDNA has proven valuable in predicting both durable responses and hyperprogression^[7]^. Extrapolating such utility to esophageal cancer demands mechanistic, ICI-specific stratification. Without it, the clinical applicability of ctDNA in modern multimodal settings remains uncertain and potentially misleading.

Finally, the reported four-fold increase in recurrence and mortality risk among ctDNA-positive patients is compelling but may be confounded by unmeasured variation in adjuvant therapy. If ctDNA-positive patients systematically received more – or less – intensified postoperative treatment, the observed prognostic effect may reflect treatment allocation rather than molecular risk^[8]^. This limitation undermines the interpretation of ctDNA as an independent predictor. Given ctDNA’s growing role in guiding adjuvant therapy in other cancers, future trials should incorporate ctDNA into randomized escalation or de-escalation frameworks to determine whether it functions as a prognostic marker or a modifiable therapeutic determinant.

Beyond its academic contribution, the study by Shen *et al* highlights systemic barriers to translating ctDNA into actionable perioperative tools. As ctDNA gains traction for adjuvant stratification, its clinical adoption remains constrained by biologic uncertainty, infrastructure inequities, reimbursement limitations, and regulatory ambiguity. Without standardized thresholds or escalation algorithms, clinicians may overtreat ctDNA-negative patients or defer necessary interventions, undermining precision. Bridging this translational gap requires policy support for registry-linked trials, real-world validation platforms, and equitable assay access. Crucially, guidelines must shift from prognostic acknowledgment to response-guiding frameworks that convert molecular signals into timely, accountable care – transforming ctDNA from a passive marker to an active driver of therapeutic coordination in surgical oncology. This correspondence aims to guide future surgical protocols and inform clinical guidelines by highlighting key translational bottlenecks and proposing system-level solutions for ctDNA-integrated care in esophageal cancer.

## Data Availability

No datasets were generated or analyzed for this article. Data sharing is not applicable.

## Research registration unique identifying number (UIN)

This study did not involve human participants; therefore, registration and a unique identifying number (UIN) were not required.
